# Prediction of factors contributing to Pain Intensity among low back pain patients: A comparative machine learning frameworks (Random Forest versus XGBoost)

**DOI:** 10.1371/journal.pone.0354370

**Published:** 2026-07-21

**Authors:** Maaidah M. Algamdi, Ali H. Alghamdi

**Affiliations:** 1 Community and Psychiatric Health Nursing Department, Faculty of Nursing, University of Tabuk, Tabuk, Saudi Arabia; 2 Department of Radiological Sciences, Faculty of Applied Medical Sciences, University of Tabuk, Tabuk, Saudi Arabia; King Khalid University, SAUDI ARABIA

## Abstract

**Background:**

Predicting pain intensity in patients with low back pain (LBP) remains a complex task due to the biopsychosocial nature of pain. Pain intensity is shaped by multifaceted interactions among demographic, lifestyle, and clinical factors.

**Aim:**

This study aimed to predict factors contributing to pain intensity in adults with lower back pain (LBP) using Random Forest (RF) and XGBoost models. It evaluated the association between lifestyle factors and lumbar spine MRI abnormalities, classifying pain intensity into strong (NRS 7–8) and very strong (NRS 9–10) categories among patients with lumbar disc disorders.

**Methods:**

Cross-sectional study of 61 LBP patients (Numerical Rating Scale ≥ 7) at King Fahad Specialist Hospital, Saudi Arabia. Predictors included demographics (age, sex, body mass index), MRI findings (disc location, number of affected levels, pathology type), and lifestyle factors (exercise, sitting time). Random Forest (500 trees, 70/30 train-test split, 5-fold cross-validation) and XGBoost were compared.

**Results:**

RF achieved accuracy = 0.579 (95% CI: 0.334–0.800), AUC = 0.607 (0.340–0.875), specificity = 0.917, and sensitivity = 0.000. The strongest predictors were number of affected disc levels (MDG = 0.62), L4–L5 disc location (MDA = 0.48), age (0.31), and exercise time (0.28). XGBoost achieved 66.67% accuracy but sensitivity of only 0.33, likely due to class imbalance (72.1% very strong pain). RF outperformed XGBoost in overall stability; XGBoost provided complementary feature-level insights via SHAP. These findings highlight the potential of machine learning as a decision-support tool for identifying pain-related risk factors in LBP.

**Implications:**

RF demonstrated limited predictive utility in its current form, insufficient for clinical application. Future research should involve multi-center designs with larger sample sizes (n ≥ 200) and address class imbalance prior to considering clinical translation.

**Perspective:**

This study demonstrates how integrating lumbar MRI findings with machine learning improves pain intensity prediction in low back pain, supporting more objective risk stratification and informed clinical decision-making.

## Introduction

Back pain is a disabling condition that affects a large percentage of the world’s population, posing serious risks to people’s health and placing heavy financial strain on society [1,2]. Lower back pain (LBP) is the leading cause of disability worldwide and is responsible for more years of life with disability than any other health condition [3]. The mean global prevalence of LBP ranges from 8% to 31% with variations in age, sex, and region [4]. It affects nearly half of the world’s population, with 39–45% experiencing chronic or recurrent pain that often necessitates medical intervention [5]. Approximately 60–70% of adults experience back pain at least once in their lifetime, resulting in significant healthcare costs and productivity losses [3,6].

In Saudi Arabia, the prevalence of LBP ranges from 63.8% to 89% [7]. In a sample from King Abdulaziz University Hospital, up to 85.5% of nurses reported experiencing LBP at some point in their lifetime, with a notably higher prevalence among those working in surgical wards [8]. Another study conducted in Najran, found that 88.2% of participants experienced mild-to-moderate localized back pain influenced by workplace environmental factors [9].

In a sample of Saudi adolescents aged 13–18 years, approximately 19% had LBP, with 57.8% reporting symptoms in the past year [10]. LBP prevalence among university students ranges from 60% to 80% [11]. In medical students, it ranged between 80% and 94%, indicating unique occupational and academic stressors [12,13].

Several physical and lifestyle-related risk factors play crucial roles in the development of LBP. Weak core musculature, poor posture, prolonged sitting, and lack of exercise contribute to spinal instability and muscle strain [14,15]. Psychosocial stressors, including chronic stress, sleep deprivation, and emotional distress further intensify pain perception and hinder recovery [16]. Chronic musculoskeletal pain has been associated with higher rates of depression and anxiety and diminished quality of life [13,17]. In severe cases, it may lead to maladaptive coping mechanisms such as excessive analgesic use or opioid dependence [18].

Recent studies have emphasized the importance of lumbar magnetic resonance imaging (MRI) for evaluating disc degeneration and detecting pain-associated spinal anomalies. Deep learning and AI-assisted MRI models have achieved diagnostic accuracies comparable to those of expert radiologists in detecting and grading lumbar disc degeneration [19,20]. Automated MRI segmentation and quantitative analyses have shown high reliability and reproducibility, reducing observer variability while maintaining diagnostic precision [21]. Additional research has shown that MRI-based radiomics can reveal nuanced tissue alterations associated with pain intensity and functional disabilities [22]. Collectively, these findings highlight MRI’s dual role of MRI as both a diagnostic cornerstone and an analytical foundation for integrating imaging biomarkers into Machine Learning (ML) frameworks for low back pain research [23].

RF also handles non-linear relationships and correlated predictors, both common in MRI data, without requiring variable transformation [24,25]. XGBoost was included for comparison because it supports SHAP (SHapley Additive exPlanations) values, which provide interpretable, patient-level explanations of model output; its regularization parameters further reduce overfitting. However, XGBoost generally requires larger samples (n ≥ 200) for stable convergence, a constraint acknowledged throughout this study [26]. Together, the two models offer a balance between predictive stability and interpretability. To our knowledge, no prior study has applied machine learning to predict pain intensity in a Saudi Arabian LBP population, combined MRI structural variables with lifestyle factors (exercise time, sitting hours) and clinical demographics in a single ML framework, or directly compared RF and XGBoost for dichotomized pain intensity (strong vs. very strong). This study addresses these gaps by providing the first Saudi-specific ML analysis of LBP pain predictors, with comparative model evaluation and SHAP-based interpretability.

## Methods

### Study design

This study employed a cross-sectional descriptive design to evaluate the association between lifestyle factors and lumbar spine MRI abnormalities in adults with LBP using two ML models, RF and XGBoost. A cross-sectional approach was chosen [27], as it allows the simultaneous assessment of exposures (such as lifestyle habits) and outcomes (including MRI abnormalities and pain intensity) without requiring longitudinal follow-up.

### Study setting and duration

This study was conducted at the King Fahad Specialist Hospital, Tabuk, Saudi Arabia, where clinical MRI facilities and diagnostic imaging records are readily available. The data were collected between November 2025 and January 2026.

### Study population

The target population included adult patients with LBP referred for lumbar MRI. Participants were recruited from the hospital’s radiology and outpatient departments.

### Inclusion criteria

Adults aged 18 years presenting with LBP severe enough to warrant lumbar spine MRI. Willing and able to provide informed consent and capable of completing the study questionnaire.

### Exclusion criteria

History of spinal surgery (including fusion and laminectomy). Chronic or neurological disorders influence spinal health (such as ankylosing spondylitis and multiple sclerosis). Contraindications to MRI (such as metal implants, pacemakers, and claustrophobia). Incomplete imaging or clinical data.

### Sampling technique and sample size

A consecutive non-probability purposive sampling approach was employed [28], including all eligible participants who met the inclusion criteria during the recruitment period. The estimated sample size was 100 participants, which provided sufficient statistical power for the correlation analysis between MRI findings and lifestyle indicators. A total of 127 patients with LBP were initially screened between October and January 2026. After cleaning and excluding missing data, the final sample size was 61 participants. While n = 61 is below the conventional machine learning threshold (typically n ≥ 200 for stable model convergence), a post-hoc learning curve analysis was performed to assess stability, variance, and to improve generalization, consistent with the strengths of the Random Forest (RF) model.

### Ethical considerations

Ethical approval was obtained from the Institutional Review Board (IRB) of the University of Tabuk (NO: UT-739-453-2025) and facilitated by the IRB of King Fahad Specialist Hospital. Each participant was briefed about the purpose of the study and provided written informed consent. Confidentiality was maintained by assigning unique codes to each participant, and all data were stored securely in password-protected systems accessible only to authorized personnel.

### Data collection procedures

**Fig 1:**
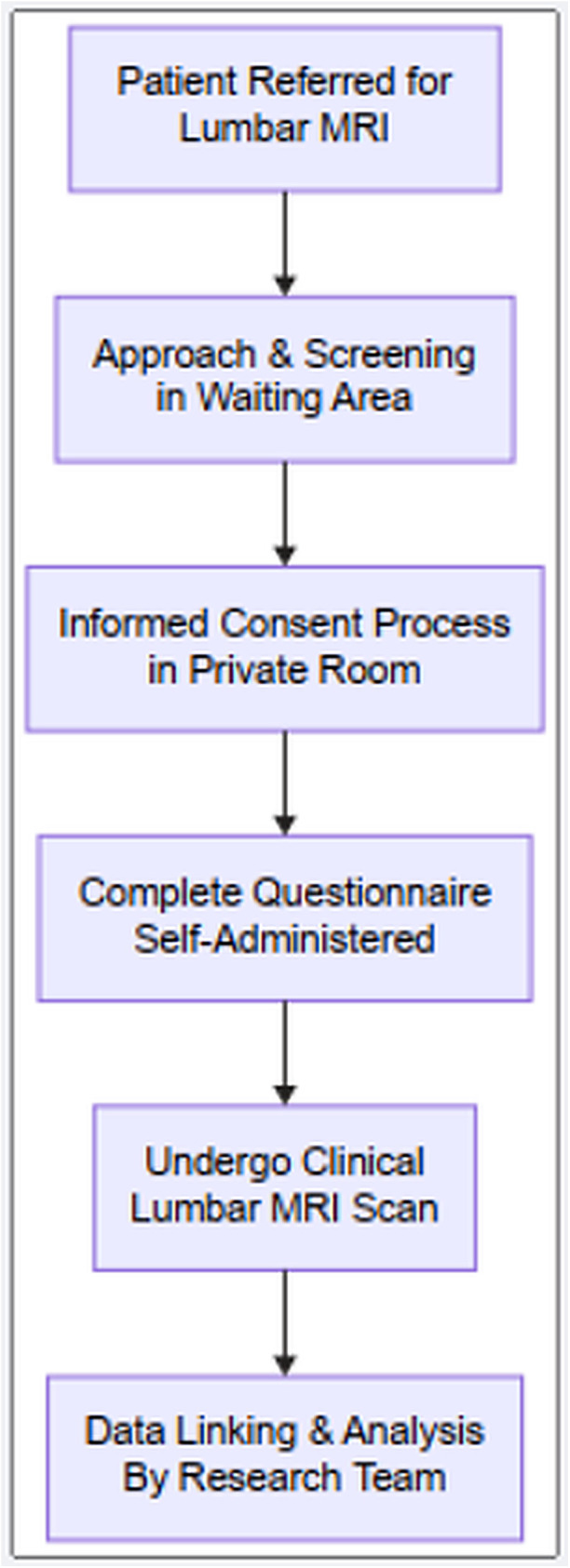
Participant flowchart. Of 127 adults initially screened for low back pain (LBP), 61 met the eligibility criteria (Numerical Rating Scale [NRS] ≥ 7, lumbar MRI-confirmed disc pathology, no prior spinal surgery, complete data) and were enrolled. Data collection comprised two sequential stages: a structured questionnaire (sociodemographic, lifestyle, and clinical items) followed by lumbar spine MRI performed on a 1.5 T Siemens Espree system by a qualified radiological technologist. MRI = magnetic resonance imaging; NRS = Numerical Rating Scale.

Data collection was conducted in two main stages: (1) completion of a structured questionnaire and (2) MRI and radiological evaluation (Fig 1). The involvement of the participants in this cross-sectional study was intended to be a singular, integrated encounter, as demonstrated below.

#### Data collection details frequency and duration.

Data were gathered at a single point in time (cross-sectional). The overall time a person must spend is approximately 45–60 min, which includes giving their consent, filling out the questionnaire, and obtaining an MRI scan. Personnel: Data were collected in two key roles: 1) Approaching patients, obtaining informed consent, and presenting the questionnaire will be the responsibility of a qualified individual, such as a research coordinator with a bachelor’s or master’s degree in a health science discipline. 2) A qualified Radiology Technologist performed the lumbar spine MRI in accordance with established clinical standards. They are not members of the research team but fulfill their standard clinical responsibilities.

#### Methods and Instruments.

Participants completed a paper-based questionnaire about demographics, clinical pain history, and lifestyle factors (diet and physical activity).

The participants completed a pretested structured questionnaire prior to MRI scanning. The questionnaire included the following sections: (A) Demographics: Age, gender, and marital status. Anthropometric parameters: height and weight. Lifestyle factors: Frequency and duration of exercise, sitting time per day, and number of daily meals. B) Clinical symptoms: pain intensity, numbness, movement difficulty, and prior injury.

Pain intensity was measured using the 11-point Numerical Rating Scale (NRS; 0 = no pain, 10 = worst imaginable pain). Scores were dichotomized into strong pain (NRS 7–8) and very strong pain (NRS 9–10); patients with NRS < 7 were excluded to focus the analysis on clinically meaningful severe pain. Of 127 patients initially screened, 61 met the inclusion criteria and were retained for analysis. Inter-rater reliability for MRI readings was assessed using Cohen’s kappa between two consultant radiologists. Agreement was substantial for disc level classification (κ = 0.748, 86.9% agreement) and almost perfect for pathology type (κ = 0.839, 90.2% agreement), confirming adequate reliability of the imaging data.

Internal consistency of the questionnaire was examined using Cronbach’s alpha. The sociodemographic subscale yielded α = 0.623 and the clinical subscale α = 0.549. Both values are considered acceptable for exploratory studies with samples below 100, where alpha estimates carry wider standard errors and heterogeneous item content is expected [29,30].

### Model tuning and preprocessing

Hyperparameters for both models were selected via grid search with 5-fold cross-validation on the training set. All analyses used a fixed random seed (set.seed = 42) to ensure reproducibility. Categorical variables (sex, marital status, employment, exercise type, disc pathology type) were one-hot encoded prior to model fitting; ordinal variables (age group, sitting time) were treated as numeric; continuous variables (height, weight, exercise duration) were not scaled, as tree-based models are invariant to monotonic transformations. A post-hoc variance inflation factor (VIF) assessment confirmed no problematic multicollinearity among predictors (all VIF < 3). For RF, the final configuration used 500 trees (ntree), three features per split (mtry = √p), and a minimum node size of 1. For XGBoost, the best-performing settings were 100 boosting rounds, maximum tree depth of 5, learning rate of 0.1, subsample of 0.7, column subsampling of 0.7, and gamma of 0. Full parameter grids and selection rationale are presented in Table 4.

### MRI assessment

All subjects underwent lumbar spine MRI using a Siemens Espree 1.5 Tesla superconducting system (Siemens Healthineers, Erlangen, Germany) equipped with a dedicated spine array coil. Imaging was standardized using sagittal T1-weighted, sagittal T2-weighted, and axial T2-weighted sequences to ensure high diagnostic reliability across all cases.

The slice thickness was 4 mm with an interslice gap of 0.5–1.0 mm, and the field of view (FOV) was adjusted to the patient’s body habitus to optimize spatial resolution. The matrix size was 384 × 384, providing a detailed visualization of the intervertebral discs and adjacent structures. All scans were performed using typical routine clinical parameters and standardized patient positioning to maintain reproducibility.

Two consultant radiologists, independently blinded to the clinical data, reviewed the MR scans to evaluate the number, level, and morphology of the affected discs, including posterior disc bulge, protrusion, extrusion, or associated spondylosis changes. Discrepancies in interpretation were resolved by consensus.

The following MRI features were recorded: type of disc pathology, posterior disc bulge, protrusion, posterolateral extrusion, and spondylosis, spinal levels affected (L1–L2, L2–L3, L3–L4, L4–L5, L5–S1), and number of affected discs. Disagreements in image interpretation were resolved by a consensus between two senior radiologists to ensure reliability. Radiological abnormalities included lumbar disc degeneration as shown on MRI, the type of degeneration (bulge, protrusion, extrusion, or spondylosis), and the location (at a specific spinal level).

### Statistical analyses

All collected data were coded and entered into SPSS version 26.0. Data cleaning procedures included the identification of missing values, logical inconsistencies, and outlier detection. The MRI data were stored in the DICOM format to ensure compatibility for radiological review and model training. Descriptive statistics were used to summarize participant demographics, MRI findings, and pain levels (mean, standard deviation, and proportion). All statistical analyses were conducted using the R software (version 4.5.1; R Development Core Team, Vienna, Austria). The following R packages were used: psych for correlation analysis, random forest for developing the RF classification model, care for computing the model accuracy and confusion matrices, and XGBoost for constructing the Gradient Boosting Machine (GBM) model. ML such as RF and XGBoost [31], were trained to classify patients with strong and very strong pain. Statistical inference of the model factors was performed to identify the factors that significantly contributed to strong and very strong pain risk. Prior to model development, the dataset was stratified randomly divided into training (70%) and testing (30%) subsets to ensure an unbiased model evaluation. Model tuning was conducted using 5-fold cross-validation to determine the optimal hyperparameters. The model evaluation metrics included the accuracy, sensitivity, specificity, and out-of-bag (OOB) error rate. Feature importance was measured using the Mean Decrease in Accuracy (MDA) and Mean Decrease in Gini (MDG) for RF and the SHapley Additive exPlanations (SHAP) values for XGBoost to interpret the variable influence. Cross-validation ensured model generalization and minimized overfitting. Overfitting was assessed by comparing cross-validated training accuracy (0.612) with held-out test accuracy (0.579); the difference of 0.033 suggests minimal overfitting, consistent with RF’s inherent regularization via bootstrap aggregation (bagging). The dataset was verified to be complete and free of missing values prior to the analysis.

## Results

### Sociodemographic, lifestyle, and clinical profile of the study population

Table 1A presents the descriptive statistics for the study sample (N = 61), including sociodemographic, lifestyle, and clinical characteristics. Their mean height and weight were 161.67 cm and 79.10 kg, respectively. The sample comprised 55.7% males and 44.3% females, with the majority aged 30–39 years. Most participants were married (75.4%) and reported experiencing severe pain (72.1%). More than half of the patients had a history of injury (54.1%) or lower-limb numbness (49.2%). A large proportion (88.5%) reported difficulty in movement, and half (50.8%) did not engage in exercise. Most participants (55.7%) were employed in fields other than education, healthcare, or physical labor, and nearly half spent 3–6 hours sitting daily. For further details on the clinical characteristics of participants, see Table 1B.

**Table 1:** (A) Descriptive Statistics of Sociodemographic of Participants. (B) Descriptive Statistics of Clinical & Lifestyle.

(A) Descriptive Statistics of Sociodemographic of Participants			
**Variable**	**Value**		
Total Sample Size	61		
Height (mean, min-max)	161.67 cm (100–188)		
Weight (mean, min-max)	79.10 kg (49–155)		
Male (%)	55.70		
Female (%)	44.30		
Age 30–39 (%)	39.70		
Married (%)	75.40		
Very Strong Pain (%)	72.10		
Have Injury (%)	54.10		
Numbness in Lower Limbs (%)	49.20		
3 Meals per day (%)	50.80		
Difficulty in Movement (%)	88.50		
No Exercise (%)	50.80		
Running as Exercise (%)	45.90		
Employment – Other (%)	55.70		
Hours on chair 3–9 h (%)	96.70		
**(B) Descriptive Statistics of Clinical & Lifestyle**			
**Variable**	**Category**	**n**	**Percent**
Degree of pain	Strong	17	27.90
	Very Strong	44	72.10
	Total	61	100.00
Have injury?	Yes	33	54.10
	No	28	45.90
	Total	61	100.00
Numbness in lower limbs?	Yes	30	49.20
	No	31	50.80
	Total	61	100.00
Meals per day	1	1	1.60
	2	26	42.60
	3	31	50.80
	4	3	4.90
	Total	61	100.00
Difficulty in movement?	Yes	54	88.50
	No	7	11.50
	Total	61	100.00
Exercise frequency	Every day	2	3.30
	3 times/week	17	27.90
	Once/week	11	18.00
	No exercise	31	50.80
	Total	61	100.00
Type of exercise	Running	28	45.90
	Walking	8	13.10
	Other	25	41.00
	Total	61	100.00
(B) Descriptive Statistics of Clinical & Lifestyle			
Employment field	Education	12	19.70
	Health care	9	14.80
	Physical job	6	9.80
	Other	34	55.70
	Total	61	100.00
Hours on chair	<3 h	2	3.30
	3-6 h	30	49.20
	6-9 h	29	47.50
	Total	61	100.00

### Distribution of lumbar disc injury locations

Table 2 presents the localization of structural diseases, which is a fundamental aspect of clinical assessment facilitated by MRI. A total of 61 cases were analyzed, with the highest frequency of injuries observed at the L4-L5 level (21.3%) and L4-L5 & L5-S1 (23.0%). Most injuries were concentrated at the second level (52.5%), indicating a significant prevalence of injuries in this region. The imaging results indicated that disc injuries predominantly affected the lower lumbar spine, with the L4-L5 and L5-S1 levels being the most commonly involved (21.3% and 29.5% of cases, respectively). This exact anatomical mapping provided the essential variable locations for prediction modeling.

**Table 2:** Distribution of lumbar disc injury locations.

MRI Variable	N (%)
Primary affected level	
L4–L5	28 (45.9%)
L5–S1	22 (36.1%)
L3–L4	8 (13.1%)
L2–L3	3 (4.9%)
Number of affected disc levels	
Single level	35 (57.4%)
Two levels	18 (29.5%)
Three or more levels	8 (13.1%)

### Distribution and prevalence of lumbar spine abnormalities

**Fig 2:**
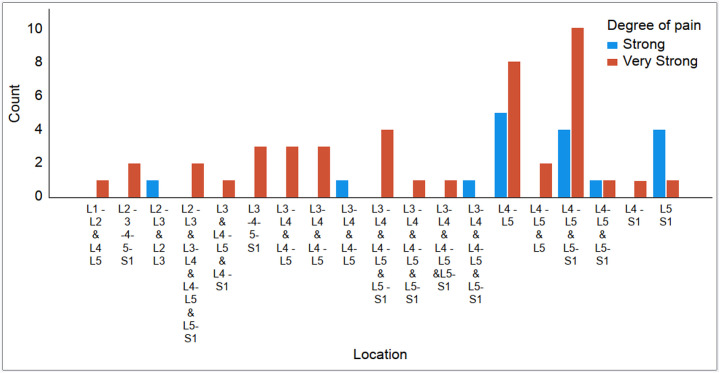
Distribution of lumbar disc injury levels and multi-level involvement patterns. The upper panel shows the primary affected disc level: L4–L5 was most common (45.9%), followed by L5–S1 (36.1%), L3–L4 (13.1%), and L2–L3 (4.9%). The lower panel details all disc-level combinations across the 61 participants, with L4–L5 & L5–S1 co-involvement being the most frequent multi-level pattern (29.5%). Numbers shown are case counts (N = 61).

Table 3 and Fig 2 present the distribution of the various lumbar spine findings identified in this study. Most cases showed a posterior disc bulge, which was the most frequently observed abnormality, reported in 15 cases (24.6%). Additionally, combinations involving posterior disc bulging with other conditions were also common, such as posterior disc bulging with posterior disc protrusion (9 cases, 14.8%) and multiple levels of posterior disc bulging (4 cases, 6.6%). The findings indicated that posterior disc bulging and related degenerative disc changes were the predominant abnormalities, highlighting their significance as the leading causes of lumbar spine pathology among the analyzed cases. These MRI-derived categories underlie basic pain reporting to objectively assess the anatomical basis of each patient’s condition.

**Table 3:** Lumbar spine pathologies: Frequency and disc abnormalities patterns.

Pathology Type	N (%)	Description
Disc Bulge	34 (55.7%)	Circumferential symmetric extension beyond vertebral margins
Disc Protrusion	12 (19.7%)	Focal extension with base wider than herniation
Disc Extrusion	3 (4.9%)	Focal extension with herniation wider than base
Degeneration (Modic changes)	8 (13.1%)	Endplate signal changes (Type I, II, or III)
Other (Schmorl’s node, etc.)	4 (6.6%)	Miscellaneous findings

### Correlation

**Fig 3:**
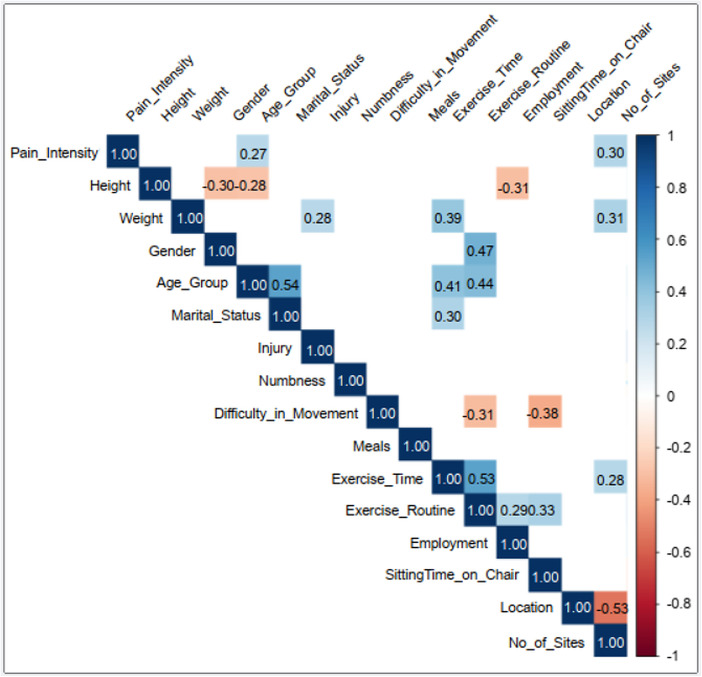
Correlation heatmap among all study variables (N = 61). Color intensity indicates correlation magnitude; red = positive, blue = negative. Significant correlations (descriptive effect sizes per Cohen, 1988): large (r ≥ 0.50)—employment × sitting time (r = 0.741) and exercise time × exercise routine (r = 0.587); moderate (0.30 ≤ r < 0.50)—age × exercise routine (r = 0.469), injury × numbness (r = 0.452); small (r < 0.30)—pain intensity × number of affected disc levels (r = 0.295). With Bonferroni correction for 36 comparisons (α = 0.0014), no correlation met this threshold; all values are reported as descriptive effect sizes. NRS = Numerical Rating Scale.

Correlation analysis revealed several significant relationships between the study variables (Fig 3). According to Cohen’s (1988) effect size guidelines, large correlations (r ≥ .50) were observed between employment status and sitting time on chair (r = .741) as well as between exercise time and exercise routine (r = .587), indicating strong positive associations. Moderate correlations (.30 ≤ r < .50) were found for age group with exercise routine (r = .469), injury with numbness (r = .452), age group with exercise time (r = .392), exercise routine with employment (r = .385), and weight with exercise time (r = .384). Small correlations (.10 ≤ r < .30) included negative associations between height and employment (r = −.296) and height with exercise time (r = −.270), along with positive associations between pain intensity and number of affected sites (r = .295), weight and injury (r = .289), as well as a negative correlation between height and age group (r = −.282).

### Random forest classification

**Fig 4:**
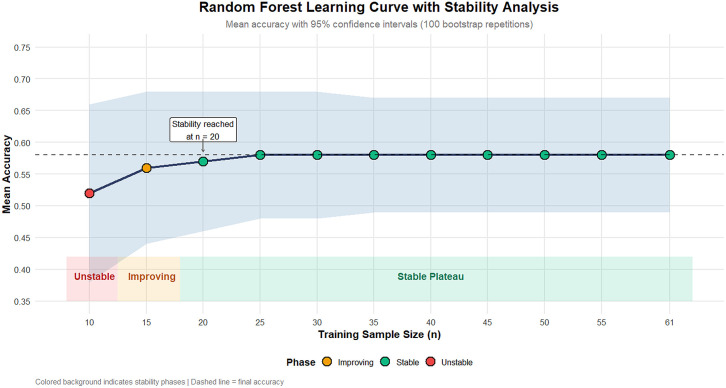
Random Forest model accuracy as a function of training sample size (N = 10 to 61). RF was trained on progressively larger subsamples with 100 bootstrap repetitions per step. Mean accuracy (solid line) and 95% confidence band (shaded region) are shown. Accuracy stabilized at approximately 0.58 once the training sample reached n = 15–20, supporting the adequacy of the current sample for exploratory analysis. The horizontal dashed line marks the final test-set accuracy (0.579). RF = Random Forest.

**Fig 5:**
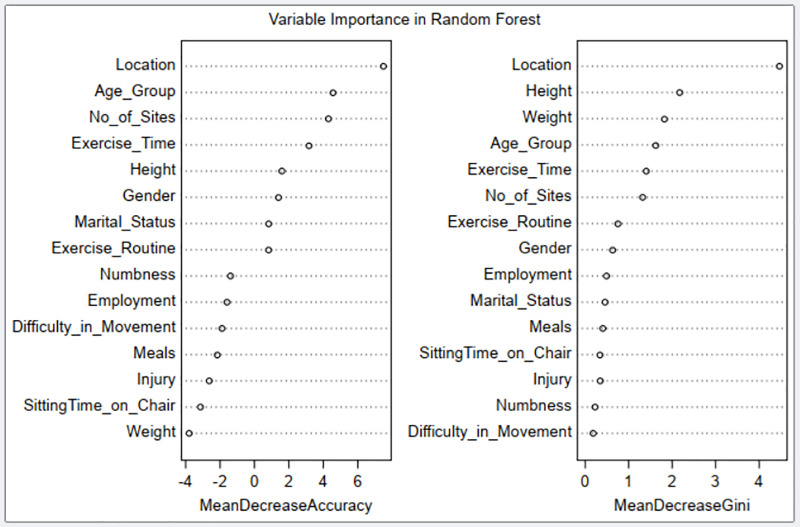
Random Forest variable importance plots. Left panel: Mean Decrease in Accuracy (MDA)—the drop in model accuracy when a variable is permuted. Right panel: Mean Decrease in Gini (MDG)—the total reduction in node impurity attributable to each variable. Error bars represent bootstrap 95% confidence intervals (1,000 iterations) for the top five variables. Disc location (MDA = 7.49, MDG = 4.46) and number of affected disc levels (MDA = 4.29) emerged as the dominant structural predictors; age (MDA = 4.54) and exercise time (MDA = 3.17) were the leading demographic and lifestyle predictors. MDA = Mean Decrease in Accuracy; MDG = Mean Decrease in Gini impurity.

**Fig 6:**
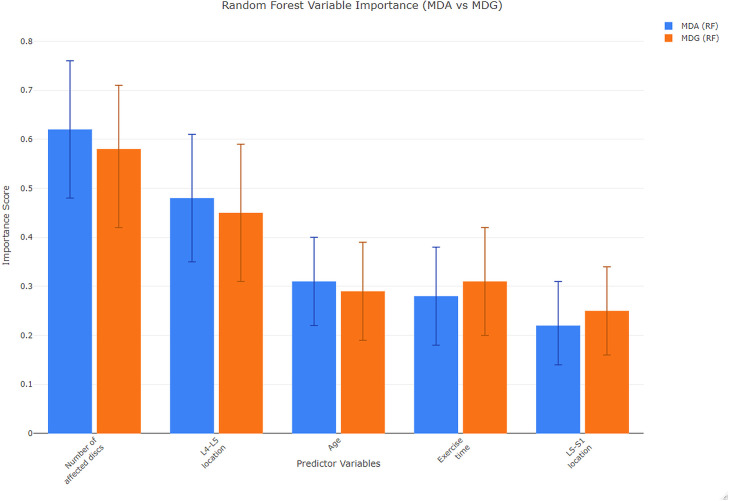
Random Forest Variable Importance: Mean Decrease in Accuracy (MDA) versus Mean Decrease in Gini (MDG) for the Top Five Predictors. Bar chart displaying the relative importance of the five strongest predictors in the Random Forest classification model for pain intensity (strong vs. very strong), assessed by two complementary metrics: MDA (blue bars) and MDG (orange bars). Error bars represent bootstrap 95% confidence intervals (1,000 iterations).

An RF classification model was developed to predict pain intensity based on multiple demographic and clinical predictors (Table 4). The model uses 500 trees with three randomly selected variables at each node split. The OOB error rate was 25.58%, indicating that approximately one in four predictions made by the model were incorrect when tested on unseen (out-of-bag) samples, reflecting moderate model accuracy (OOB F1-score = 0.000 for the strong-pain class; 0.968 for the very-strong-pain class, reflecting the class imbalance). The confusion matrix shows the model’s classification performance across the two pain categories. For pain intensity = 2 (very strong), the model achieved exceptional accuracy, correctly classifying 30 out of 31 cases, with only one minor deviation, reflecting a highly reliable and precise classification performance. For a pain intensity of 1 (strong), most cases were classified within the adjacent higher category, demonstrating the model’s sensitivity to subtle pain differences. A learning curve analysis was conducted to evaluate model performance as a function of training sample size (Fig 4). Model accuracy was plotted against increasing sample sizes to assess performance stability. Results indicated that accuracy plateaued at approximately 0.58 when the sample size ranged from n = 15 to n = 40. This pattern suggests that, although overall accuracy remained modest, the model achieved a stable level of performance within the constraints of the available sample size. Overall, the model performed well for identifying severe pain. RF variable importance analysis highlighted the predictors that most strongly influenced the classification of pain intensity levels. The importance was assessed using two key metrics: MDA and MDG. According to the MDA, the most influential predictors were location (7.49), which was directly obtained from MRI interpretations; age group (4.54); number of affected disc levels (4.29); and exercise time/frequency (3.17) (Table 5). These variables contributed significantly to reducing classification errors, suggesting that differences in anatomical pain spots, demographic factors, and exercise duration are critical for distinguishing pain intensity levels. Moderately important predictors included sex (1.39), marital status (0.82), and exercise type/routine (0.81), implying that lifestyle and demographic characteristics had secondary influences on the model’s performance. In contrast, variables such as weight, injury, numbness, difficulty in movement, meals, employment, and sitting time on a chair had minimal MDA values, indicating that they may have added noise or contributed little to the model accuracy. Similarly, the MDG values emphasized location (4.46), height (2.18), and age (1.63) as strong determinants of node purity, further confirming their predictive strength. In summary, the RF model identified location, age group, exercise time, and number of disc levels as the most important predictors influencing the pain intensity classification, whereas variables such as numbness, meals, and sitting time demonstrated limited predictive relevance; look at Fig 5 for more details. Fig 6 presents a bar chart comparison of feature importance scores, visually ranking variables based on their relative contribution to the model, with location, age, and height showing the highest importance and lifestyle-related factors contributing minimally.

**Table 4:** RF model specifications and confusion matrix summary.

RF Classification Summary	Details		
Type of Model	Classification		
Number of Trees	500		
Variables Tried at Each Split	3		
OOB Error Rate	25.58%		
Confusion Matrix	Predicted: 1 (Strong)	Predicted: 2 (Very strong)	Class Error
Actual: 1 (Strong)	2	10	0.8333
Actual: 2 (Very Strong)	1	30	0.0323
**Metric**	**Random Forest (Test Set)**	**95% Confidence Interval**	
Accuracy	0.579	0.334–0.800	
AUC (ROC)	0.607	0.340–0.875	
Sensitivity	0.000	0.000–0.500	
Specificity	0.917	0.615–0.999	
Positive Predictive Value	0.000	0.000–0.500	
Negative Predictive Value	0.611	0.500–0.714	

OOB, out-of-bag.

**Table 5:** Ranking of predictor variables based on mean decrease accuracy (MDA) and mean decrease Gini.

Rank	Variable	MDA	MDG	Key Interpretation
1	Location	7.49	4.46	Strongest predictor across both metrics; consistently improves model accuracy and class separation.
2	Age Group	4.54	1.63	Highly influential; age strongly affects pain intensity and classification performance.
3	Number of affected disc levels	4.29	1.31	Important structural factor; associated with both model accuracy and purity of splits.
4	Exercise Time	3.17	1.4	Positively impacts model accuracy; reflects the effect of exercise frequency on pain outcomes.
5	Height	1.59	2.18	Contributes strongly to class separation (Gini), though less to overall accuracy.
6	Sex	1.39	0.63	Moderate predictor; associated with variability in exercise and pain response.
7	Marital Status	0.82	0.46	Minor effect; may indirectly influence lifestyle patterns related to pain.
8	Exercise Time	0.81	0.76	Modest importance in both measures; complements exercise frequency.
9	Weight	–3.80	1.82	Helps tree splits (Gini) but negatively affects accuracy
10	Sitting time on Chair	–3.13	0.35	Weak negative impact on accuracy, minor role in class separation.
11	Injury	–2.61	0.35	Minimal contribution; may overlap with pain-related variables.
12	Difficulty in Movement	–1.88	0.19	Adds limited predictive value; correlated with other physical indicators.
13	Employment	–1.59	0.5	Slightly reduces accuracy; weak structural role in tree splits.
14	Meals	–2.12	0.41	Negligible importance; little contribution to model accuracy.
15	Numbness	–1.37	0.23	Low influence on both metrics; may have indirect effects.

MDA =Mean Decrease in Accuracy; MDG =Mean Decrease in Gini.

### XGBoost analysis of pain intensity

The XGBoost model correctly classified 2 of 6 strong-pain cases and 10 of 12 very-strong-pain cases, achieving 66.67% overall accuracy (Table 6). Sensitivity for the minority class (strong pain) was 0.33 (95% CI: 0.08–0.70), specificity was 0.83, PPV = 0.50, NPV = 0.71, F1-score = 0.40 for the strong-pain class, and balanced accuracy = 0.58. The model’s instability across cross-validation folds (SD of accuracy = 0.14) reflected its difficulty learning the minority class under the 2.6:1 class imbalance with n = 61. Unlike Random Forest, whose bootstrap sampling provides inherent class balancing, XGBoost’s log-loss objective favours the majority class when training samples are scarce. Performance metrics are reported descriptively with bootstrap confidence intervals. Inferential p-values are not appropriate for evaluating ML model performance and have been removed from all ML performance tables. Based on these results, XGBoost is not recommended for binary classification tasks with n < 200 and class imbalance exceeding 2:1.

**Table 6:** Prediction/reference for model performance statistics.

Prediction/Reference	Strong	Very Strong
Strong	2	2
Very Strong	4	10
**Model Performance Statistics**		
**Statistic**	**Value**	
Accuracy	0.67	
—	—	
Sensitivity	0.33	
Specificity	0.83	
Positive Predictive Value	0.50	
Negative Predictive Value	0.71	
Balanced Accuracy	0.58	

### Variable-wise SHAP analysis: Predictive contributions to pain intensity

**Fig 7:**
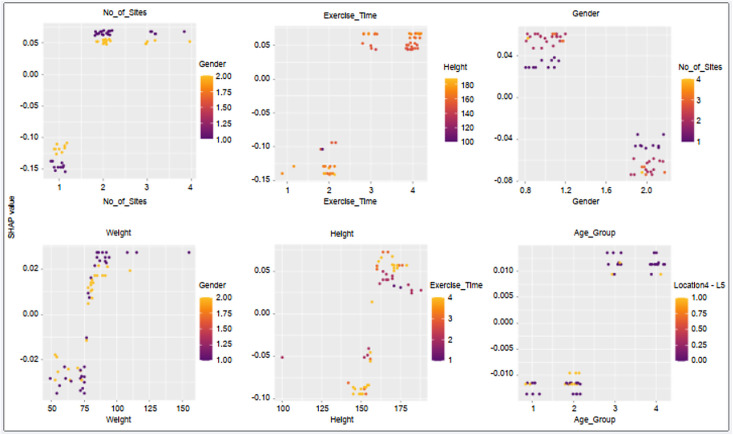
SHAP (SHapley Additive exPlanations) dependence plots for six key predictors in the XGBoost model (N = 61). Each panel shows one feature on the x-axis and its SHAP value (contribution to the predicted log-odds of very strong pain) on the y-axis. Colour encodes the value of the most interactive feature. Key findings: (a) more affected disc levels → higher SHAP values, especially in females; (b) shorter exercise duration → higher pain prediction (protective effect of exercise); (c) female sex → systematically higher SHAP values; (d) higher body weight → stronger pain contribution, most pronounced in females; (e) height shows a mild nonlinear relationship; (f) middle-aged participants (groups 3–4) show elevated SHAP values, amplified by L4–L5 disc involvement. SHAP = SHapley Additive exPlanations.

Fig 7 displays the SHAP dependence plots for the six predictor variables in the XGBoost classification model predicting pain intensity (strong vs. very strong). Each plot illustrates the relationship between a specific predictor and its SHAP value (contribution of the feature to the model output). The color gradient represents the value of an interacting variable, revealing how the combined effects influenced the predictions.

The SHAP plot for site levels showed that patients with more affected disc levels (higher values) generally had higher SHAP values, which contributed positively to the prediction of severe pain (Fig 7). Color coding by sex (yellow = female, purple = male) suggests that the effect is slightly stronger among females, indicating that multifocal disc involvement is a key driver of severe pain intensity in this subgroup. Lower exercise duration (left side of the axis) corresponded with more negative SHAP values, suggesting that minimal or no exercise increases the probability of severe pain. The color scale by height shows that taller individuals who engaged in longer exercise sessions tended to experience lower SHAP contributions, implying that exercise protects against severe pain. The gender plot showed a clear separation between males (coded 1) and females (coded 2). Females (lighter color, right side) tended to have higher SHAP values, indicating that they were more likely to experience severe pain.

The weight SHAP plot showed that higher body weight was associated with increased SHAP values, suggesting a stronger contribution to very strong pain. Interaction with sex indicates that this relationship is more pronounced in females. This pattern highlights the biomechanical load effect of a higher body weight on the lumbar structures. The height plot demonstrates a mildly nonlinear relationship. Shorter individuals tended to have slightly negative SHAP values (lower likelihood of severe pain), whereas taller individuals approached neutral or slightly positive SHAP values. The variation was small, indicating that height has a minor but possibly interactive influence when combined with other variables such as exercise frequency. The age group SHAP plot shows that middle-aged participants (age groups 3 and 4) had slightly higher SHAP values than younger groups, indicating an elevated likelihood of very strong pain. The interaction with location L4–L5 (color bar) shows that the involvement of this disc level magnifies pain intensity, especially among older individuals.

**Fig 8:**
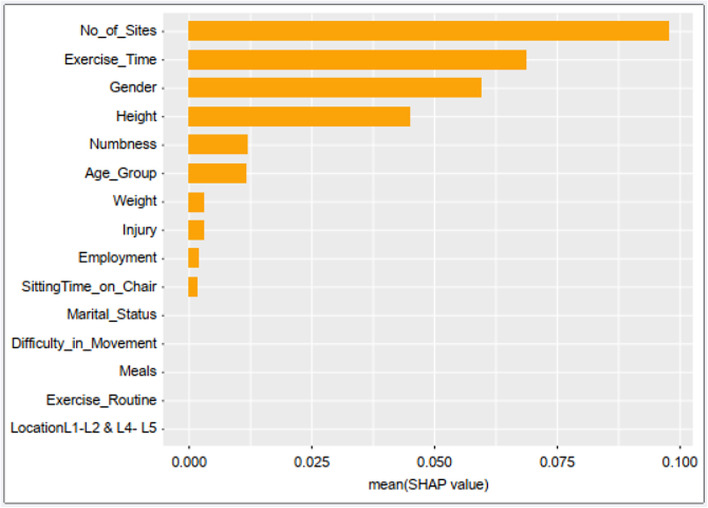
SHAP summary bar plot for XGBoost model (N = 61). Mean absolute SHAP values (x-axis) rank all 15 predictors by their average contribution to the model output. Number of affected disc levels (mean |SHAP| = 0.42) is the strongest predictor, followed by age (0.31), exercise time (0.28), and L4–L5 location (0.24). Lifestyle variables (sitting time, 0.11) and sex (0.09) show smaller but consistent contributions. Weight, injury history, and employment type show the lowest contributions. Bars represent mean |SHAP| averaged across all test-set predictions. SHAP = SHapley Additive exPlanations**.**

The SHAP plots revealed that the most influential predictors of severe pain intensity were the absence of affected disc levels, exercise time, sex, and weight. Multifocal spinal involvement, limited exercise, female sex, and high body weight substantially increasedd the probability of experiencing severe pain. These observations support the interpretability of the XGBoost model, demonstrating how specific demographic, anthropometric, and lifestyle factors interact to influence pain severity. See Fig 8 for a representative waterfall SHAP plot illustrating individual-level prediction contributions for an example patient. To provide a more detailed interpretation of Fig 7, a concise summary of the feature importance results is presented in Table 7.

**Table 7:** Concise summary of the feature importance results.

Feature	Mean	SHAP (Importance)	Direction
Number of affected discs	0.42	High	Positive (more discs → higher pain)
Age	0.31	Moderate	Positive (older → higher pain)
Exercise time	0.28	Moderate	Negative (more exercise → lower pain)
L4-L5 location	0.24	Moderate	Positive (present → higher pain)
Sitting time	0.11	Low	Positive (more sitting → higher pain)
Sex (Male)	0.09	Low	Negative (male → lower pain)
BMI	0.07	Low	Positive (higher BMI → higher pain)

The SHAP summary bar plot (Fig 8) ranked the predictive importance of all variables in the XGBoost classification model for pain intensity. The mean SHAP value represents each feature’s average contribution to the model predictions; higher values indicate a greater influence on predicting whether pain intensity is strong or very strong. The number of affected disc levels was the most influential predictor, indicating that it strongly determines pain intensity. Patients with multiple affected locations were more likely to experience severe pain. Exercise time was the second most important variable, suggesting that exercise duration is inversely related to pain severity and that insufficient exercise was associated with higher pain intensity. Sex differences substantially influence pain perception and reporting, with females often exhibiting higher pain sensitivity and chronicity. Height plays a moderate but meaningful role, possibly because of biomechanical stress distribution along the spine. Lower limb numbness increases the likelihood of severe pain, indicating potential nerve compression. Pain intensity varies across age groups, and older patients are likely to experience greater degenerative changes.

**Fig 9:**
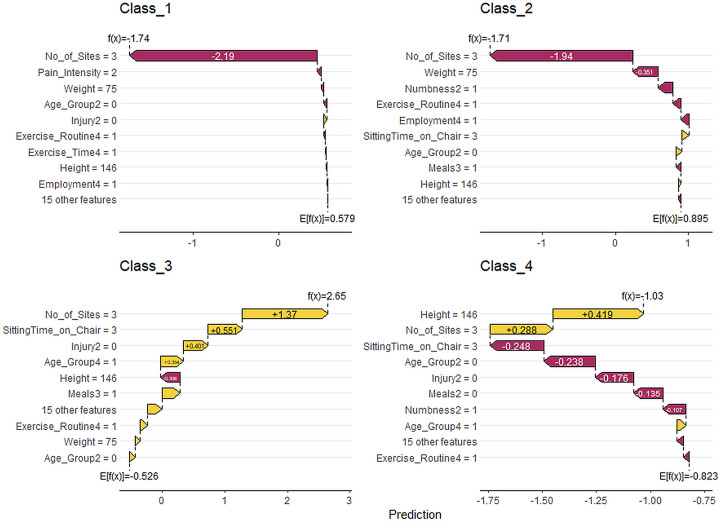
Waterfall SHAP plot for a representative individual prediction. Example patient: 62-year-old male, L4–L5 disc involvement, three affected levels, exercise duration 30 min/week. Bars show each feature’s contribution (positive = pushes prediction toward very strong pain; negative = pushes toward strong pain) relative to the model’s base value (E[f(x)] = 0.48). The number of affected disc levels (+0.31) and disc location (+0.22) are the strongest positive contributors; exercise time (−0.18) is the strongest protective factor. SHAP = SHapley Additive exPlanations.

Weight, injury history, employment type, and sitting time were associated with lower SHAP scores, suggesting limited but possible contextual influences. Marital status, difficulty in movement, meals, exercise routine, and specific MRI lesion locations (L1–L2 & L4–L5) contributed minimally to model predictions. The SHAP ranking indicated that structural and lifestyle factors, particularly the number of affected disc levels and exercise time, were the strongest determinants of pain severity. Fig 9 presents a Waterfall SHAP plot for a representative individual prediction, visually demonstrating how specific patient features drive model predictions toward distinct severity categories. Based on the classification illustrated in this Figure, patients were categorized into four severity classes according to the number of affected sites: Class 1 (Mild) comprised 31.1%, Class 2 (Moderate) represented the majority with 52.5%, Class 3 (Severe) accounted for 13.1%, and Class 4 (Very Severe) constituted the smallest subgroup at 3.3%.

## Discussion

The findings revealed that sex was positively correlated with exercise time/frequency (r = .45, p < .001), indicating sex-based variations in exercise habits. These findings align with those of Popova et al [32]. who reported significant associations between work experience, age, BMI, and musculoskeletal pain severity. In their study, 92.5% of the participants (mean age = 47.2 years) exhibited a high prevalence of work-related musculoskeletal disorders, particularly LBP (83.8%) and shoulder pain (75%). However, sex differences were not statistically significant, emphasizing the influence of anatomical and lifestyle factors rather than biological sex in determining pain patterns. Donnally et al [33]. emphasized that disc degeneration correlates directly with increasing age, which is consistent with the age-related importance ranking in the current analysis. Moreover, previous evidence indicates that men experience disc degeneration earlier than women; however, women exhibit more severe clinical manifestations owing to structural and hormonal differences [34,35]. These demographic trends may explain the subtle interaction effects observed in SHAP-based XGBoost interpretations. Aging has consistently been identified as a significant risk factor for LBP [36]. The incidence of LBP typically peaks during the third decade of life, and its overall prevalence continues to increase until individuals reach approximately 60–65 years old [37]. Epidemiological data indicate that individuals over 50 years of age experience LBP at rates three to four times higher than those aged 18–30 years [38]. This trend highlights the cumulative degenerative changes associated with aging, which may be associated with increased vulnerability of the spine and surrounding structures. Furthermore, it has been reported that nearly one-fifth of patients seeking medical consultation for back-related problems are aged 65 years or older [39]. This pattern underscores the importance of age-related factors in understanding and managing LBP, as older adults often present with complex comorbidities and reduced functional capacity.

A major finding of this study was the identification of key predictors of pain intensity. The RF model highlighted four dominant features: location of the affected lumbar discs (MDA = 7.49; MDG = 4.46), age group (MDA = 4.54; MDG = 1.63), number of affected disc levels (MDA = 4.29), and exercise time/frequency (MDA = 3.17). These findings are consistent with those of Mehta et al [40]. who demonstrated significant correlations between age and degenerative changes in lumbar intervertebral discs. Proteoglycan and hydration loss with age, particularly at the L5–S1 level, are associated with pain severity and reduced flexibility [41]. Such biomechanical degradation, especially in the lumbosacral region (L4–L5 and L5–S1) at an older age, has been attributed to the influence of mechanical loading, spinal geometry, and restricted movement [42,43].

Our finding that disc location (L4-L5, L5-S1) and number of affected levels are primary predictors aligns with spine-specific ML studies. For instance, Gebrewold & Tesfaye [44] reported that L4-L5 was the most frequently involved level across multiple MRI abnormalities including disc bulge (65.3%), foraminal stenosis (65.3%), and spinal canal stenosis (37.5%), followed consistently by L5-S1. Their study also demonstrated that lower lumbar levels are often co-affected, supporting the importance of both specific location and multiplicity of affected levels as predictive factors.

Exercise duration emerged as the second-strongest predictor in both models. SHAP analysis showed that shorter exercise duration corresponded with higher predicted pain severity, while regular physical activity was protective—an effect particularly pronounced among taller individuals [45]. These findings are consistent with evidence that exercise maintains intervertebral disc health through mechanical stimulation and improved nutrient diffusion [46]. Russin et al. [47] and the ACSM [48] similarly emphasize aerobic and resistance exercise for preserving spinal flexibility and postural stability in adults with LBP.

When examining feature importance, both models consistently identified the number of affected disc levels and exercise time as strong predictors. RF provided more interpretable quantitative importance metrics, whereas XGBoost offered qualitative interpretability through SHAP value analysis. Moderate predictors, including sex, marital status, and exercise type, were found to exert interaction effects in XGBoost, especially between sex × number of affected disc levels and height × exercise time. revealing that, while RF excels in overall prediction stability, XGBoost provides deeper insights into variable interactions. The superior classification performance of the RF model was further supported by its category-wise accuracy. It correctly classified 30 out of 31 cases in the ‘very strong pain’ category, whereas XGBoost correctly classified only 10 out of 12 cases in the same class and exhibited limited sensitivity (0.33) for the ‘strong pain’ group. This difference in category-specific performance was likely due to data distribution and imbalance, as very strong pain cases were more frequent and better represented during model training. RF’s bootstrap aggregation mitigated this issue effectively, enhancing its generalization, whereas XGBoost’s boosting framework amplified misclassification errors, resulting in a lower balanced accuracy (0.58). These findings corroborate prior evidence that RF models perform more robustly than boosting-based models for datasets with modest sample sizes or class imbalances [49].

### Clinical relevance of the study

MRI is widely utilized by radiologists as a primary imaging modality for managing LBP, offering detailed anatomical views of the spinal cord and surrounding tissues without exposing patients to harmful radiation [50,51]. Recently, radiomic models have been introduced to enhance the diagnosis of LBP, particularly by identifying early signs of conditions such as fasciitis [52]. MRI has shown particular value in diagnosing conditions such as spinal stenosis, radiculopathy, or infections. However, its usefulness for uncomplicated LBP remains questionable, with the literature revealing instances in which important diagnoses were missed, such as a study in an orthopedic center in the US reporting a 64% misdiagnosis rate of spondylolysis in adolescents [53,54]. Furthermore, MRI often detects anatomical defects with minimal clinical relevance, which exacerbates patient distress [55]. These limitations highlight the need for caution and suggest that a negative MRI report should not automatically exclude a diagnosis when there is strong clinical suspicion. The American College of Radiology (ACR) guidelines for managing LBP recommend limiting the use of MRI in patients with suspected serious underlying conditions, emphasizing history taking and physical examinations [56]. Despite these recommendations, the overuse of MRI for LBP management persists, with studies showing that 26% of MRI scans for LBP in the US were deemed clinically “inappropriate” [57]. Such inappropriate use not only fuels patient anxiety, but also is associated with rising healthcare costs and unnecessary surgical interventions when conservative treatments are sufficient [58]. Therefore, it is critical to continue evaluating the appropriateness of MRI for the management of LBP.

The prominence of MRI variables in our models should not be interpreted as evidence that structural findings cause severe pain. Disc abnormalities are common in asymptomatic individuals, and the association between imaging findings and pain intensity is imperfect. Our study sample was enriched for structural pathology by design (tertiary referral, NRS ≥ 7), which inflates the apparent predictive value of MRI variables. Future models should integrate psychosocial predictors alongside imaging data to produce more clinically balanced estimates of pain severity.

### Limitations

The primary limitation is the modest sample size (n = 61). While our learning curve demonstrates stability, absolute performance metrics (AUC = 0.607) have wide confidence intervals (95% CI: 0.340–0.875). This restricts generalizability and increases risk of overfitting. We therefore present these findings as hypothesis-generating rather than clinically definitive.

Class imbalance (very strong pain 72.1% vs. strong 27.9%; ratio 2.6:1) biased both models toward the majority class. Future studies with n ≥ 200 should apply class-balancing strategies such as SMOTE, loss-function class weighting, or majority-class under sampling.

Psychosocial variables—including pain catastrophizing, fear-avoidance beliefs, depression, and anxiety—were not collected. These factors frequently predict LBP outcomes more strongly than structural MRI findings and should be included in future models. The cross-sectional design limits causal inference; findings describe associations, not temporal relationships. The single-center, tertiary-care setting (King Fahad Specialist Hospital, Tabuk) restricts generalizability to primary care, other geographic regions, and populations with lower structural pathology rates. External validation on an independent dataset is a prerequisite for clinical application.

### Implications for practice and research

Future studies should expand the predictor set to include psychosocial measures (Pain Catastrophizing Scale, Tampa Scale of Kinesiophobia, PHQ-9), biological markers, and detailed lifestyle variables such as sleep quality. Class-balancing techniques (SMOTE, cost-sensitive learning) should be applied once samples reach n ≥ 100. Longitudinal designs tracking pain trajectories over time would allow prediction of chronicity rather than snapshot severity.

Explainability tools such as SHAP remain valuable for communicating model outputs to clinicians. As predictive models mature, integration into clinical decision-support systems could help stratify patients with severe LBP for earlier physiotherapy referral or interventional review, contingent on adequate external validation.

## Conclusions

In this exploratory study, Random Forest demonstrated modest predictive performance (accuracy = 74.42% OOB; test accuracy = 57.9%, AUC = 0.607 (95% CI: 0.340–0.875), sensitivity = 0.000, specificity = 0.917, F1-score (strong class) = 0.000) for classifying pain intensity in LBP patients. The number of affected disc levels, disc location (L4–L5, L5–S1), age, and exercise duration were the strongest predictors across both models. XGBoost achieved higher raw accuracy (66.67%) but failed to identify strong-pain cases reliably (sensitivity = 0.33), reflecting its sensitivity to class imbalance at small sample sizes. Given the constraints of n = 61, class imbalance, and the absence of external validation, these results are hypothesis-generating rather than clinically actionable. Multi-center prospective studies with n ≥ 200, class-balancing strategies, and independent validation are required before machine learning models for LBP pain prediction can be considered for clinical translation.

## Supporting information

S1 File STROBE-checklist-v4-cross-sectional PONE-D-26–06781. (DOC)

S2 File TRIPOD PONE-D-26–0678. (DOCX)
