# Supplementary material for: Prediction of factors contributing to Pain Intensity among low back pain patients: A comparative machine learning frameworks (Random Forest versus XGBoost)
Source: PLoS One. 2026 Jul 21;21(7):e0354370. doi: 10.1371/journal.pone.0354370 (PMC13387577; doi:10.1371/journal.pone.0354370)
Supplement: S2 File — (DOCX) [file pone.0354370.s002.docx]

TRIPOD-AI Checklist: PONE-D-26-06781

| **Section** | **Item** | **Reported** |
| --- | --- | --- |
| Title | Identify as prediction model (AI/ML) | ✓ |
| Abstract | Summarize objectives, methods, results | ✓ |
| Introduction | Explain rationale for prediction model | ✓ |
| Methods – Participants | Eligibility criteria, recruitment period | ✓ |
| Methods – Outcome | Definition, measurement, timing | ✓ |
| Methods – Predictors | Demographics, MRI, lifestyle variables | ✓ |
| Methods – Sample size | n=61, justification via learning curve | ✓ |
| Methods – Missing data | No missing (complete cases) | ✓ |
| Methods – Model development | RF (500 trees), 70/30 split, 5-fold CV | ✓ |
| Methods – Model evaluation | Accuracy, AUC, sensitivity, specificity, 95% CI | ✓ |
| Results – Participants | Flow diagram (Figure 1), characteristics (Table 1) | ✓ |
| Results – Model performance | Tables 4-5, Figures 2-3 | ✓ |
| Discussion – Limitations | Sample size, imbalance, external validation | ✓ |
| Discussion – Interpretation | Exploratory, not clinically ready | ✓ |
| STROBE Elements added:  Study design clearly stated as cross-sectional (Methods)  Report of numbers at each stage (Figure 1 flowchart)  Discussion of generalizability (Limitations section)  Location: Supplementary Material (TRIPOD-AI checklist);  Methods section (STROBE elements) | | |
